# Biological Effect of Quercetin in Repairing Brain Damage and Cerebral Changes in Rats: Molecular Docking and In Vivo Studies

**DOI:** 10.1155/2022/8962149

**Published:** 2022-04-26

**Authors:** Ahmed B. M. Mehany, Amany Belal, Eman Y. Santali, Salwa Shaaban, Mohammad A. S. Abourehab, Ola A. El-Feky, Mahmoud Diab, Fawzy M. A. Abou Galala, Eslam B. Elkaeed, Ghada Abdelhamid

**Affiliations:** ^1^Department of Zoology, Faculty of Science, Al-Azhar University, Nasr City, Cairo, Egypt; ^2^Medicinal Chemistry Department, Faculty of Pharmacy, Beni-Suef University, Beni-Suef 62514, Egypt; ^3^Department of Pharmaceutical Chemistry, College of Pharmacy, Taif University, P. O. Box 11099, Taif 21944, Saudi Arabia; ^4^Department of Microbiology & Immunology, Faculty of Pharmacy, Beni-Suef University, Beni-Suef, Egypt; ^5^Department of Clinical Laboratory Sciences, Faculty of Applied Medical Sciences, King Khalid University, Abha, Saudi Arabia; ^6^Department of Pharmaceutics, Faculty of Pharmacy, Umm Al-Qura University, Makkah 21955, Saudi Arabia; ^7^Department of Pharmaceutics and Industrial Pharmacy, College of Pharmacy, Minia University, Minia 61519, Egypt; ^8^Biochemistry Department, Faculty of Pharmacy, Tanta University, Tanta 3111, Egypt; ^9^Anatomy and Embryology Department, Faculty of Medicine, Al-Azhar University, Cairo, Egypt; ^10^Department of Anatomy, Faculty of Medicine, Damita, Al-Azhar University, Cairo, Egypt; ^11^Department of Pharmaceutical Sciences, College of Pharmacy, AlMaarefa University, Riyadh 13713, Saudi Arabia; ^12^Department of Pharmacology and Toxicology, Faculty of Pharmacy, Helwan University, Egypt

## Abstract

This study examined the protective effect of quercetin against high-altitude-induced brain damage in rats. A molecular docking study was performed to investigate the potential effect of quercetin in reducing brain damages through its ability to target the oxidative stress enzymes. Biomarker assessment screening assays were also performed then followed by in vivo studies. Three groups of rats were divided into the control group, an untreated animal model group with induced brain damage, and finally, the quercetin treated group that received quercetin dose equal to 20 mg/kg of their body weights. Molecular docking studies and biomarker assessment screening assays proved the potential effect of quercetin to affect the level of representative biomarkers glutathione (GSH), glutathione reductase (GR), glutathione-S-transferase (GST), glutathione peroxidase (GPx), superoxide dismutase (SOD), catalase (CAT), and malondialdehyde (MDA). Additionally, the protective effect of quercetin against high altitude, low pressure, and low oxygen was also investigated by exploring the brain histopathology of experimental rats. Brain damage was observed in the untreated animal model group. After treatment with quercetin, the cerebral edema in the brain tissues was improved significantly, confirming the protective effects of quercetin. Therefore, quercetin can be used as a natural food additive to protect from the highaltitude-induced brain damage.

## 1. Introduction

Mountains cover over twenty percent of the Earth's surface; also, mountain climbing is a goal for many people. Those people who live or deal with a high-altitude environment are subjected to physiological changes. Furthermore, there are large numbers of people who live in locations at high altitude and they were exposed to develop acute mountain disorder [1]. The main reason of brain damage and cerebral changes is the decrease in the flow of blood to the brain cells, which causes the lack of nutrients as well as oxygen, and this deficiency leads to a deficiency in the brain as well, which results in brain damage and also results in many neurological diseases including disorders of consciousness [2]. People who live in high altitude are suffering from oxidative stress that affects their body, and they may become susceptible to changes in all cells of the body, such as acute brain injury [3]. Cerebral edema effects on these lives of such people are threatening [4]. Contrastingly, high-altitude cerebral edema can develop and cause high-altitude pulmonary edema [5]. More studies have demonstrated that free radicals resulting from high-altitude cerebral edema may lead to fluid accumulation in the brain [6]. Brain dysfunction can occur when there is an error in the blood supply to the brain. Therefore, decreasing the oxygen supply to the brain may lead to the death of brain cells. Brain ischemia induces numerous pathological events, such as programmed cell death, cytotoxicity, and brain edema [7]. Brain damage is the most common disorder that leads to death and causes disability in old age [8]. High-altitude hypoxia is considered a common health disorder that results from different human activities, such as traveling and mountaineering. It happens because of a decrease in oxygen pressure, which leads to a decrease in oxygen supply to the tissues [9]. High-altitude environments are known to have a lower oxygen pressure relative to sea level [10]. High-altitude cerebral edema is believed to be a cause of life threatening and occurs as a result of going to high-altitude environments [11].

Quercetin is found in many plants and fruits [12] and has shown diverse biological activities [13]. Furthermore, previous biomarker assessment studies have illustrated that quercetin causes an increase in the proliferation of neuron cells [14]. Also, quercetin is very important for the inhibition of some oxidative enzymes within cells and these enzymes work mainly in brain functions, which proved that quercetin has the ability to cross the blood-brain barriers and it also has a role in modifying the antioxidant pathways; for these reasons, quercetin has the ability to respond to oxidative stress induced by nervous stress. Quercetin has shown to reduce brain diseases such as Alzheimer's disease [15]. On the other hand, quercetin has potent neuroprotective and memory enhancement in humans and animals as well. The neuroprotective effects of quercetin on hippocampal and cortical neurodegeneration. The protective role of quercetin includes suppression of ROS, lipid peroxides, and production of cytokines that control the oxidative stress [16]. It has been demonstrated that quercetin plays a vital and significant role in supplying blood to the blood-brain barrier and eliminating oxidative stress. Recent studies have established the fact that quercetin is a sufficiently strong antioxidant and has significant protective effects against oxidative damage [17]. It has also been reported that quercetin has therapeutic potential and can act as a neuroprotective in cases of brain trauma [18]. Quercetin has strong antioxidant activities against free radicals [19] and can reduce lipid peroxidation [20]. Moreover, quercetin has also been proven to upregulate antioxidant levels [21]. Many people like to face health problems through natural products; some believe it has less side effects; also, there are many reported studies that focus on natural products and their role in protecting against many health problems [22–26]. All these facts encouraged us to focus on quercetin as a crucial natural product and investigate it through different aspects as molecular docking studies to explore its binding mode and affinity to the desired molecular targets, in vitro screening assay against panel of biological biomarkers and through in vivo studies also to get better insights from different investigating pathways.

## 2. Materials and Methods

### 2.1. Molecular Docking Studies

#### 2.1.1. Protein, Ligand Preparation, and Site Detection

The crystal structures for the target enzymes such as (GR, GPx, GST, SOD, and CAT) were retrieved from Protein Data Bank (https://www.rcsb.org/) as PDB files, codes are 1BWC, 10GS, 2F8A, 1AP5, and 1DGB, respectively. After downloading, these crystal structures were refined, water chains were removed, then protonation of the 3D structure was performed, and energy minimization was done by using Moe 2008.10 software. Binding sites were also detected by Moe through alpha site finder; then, the files were saved as Moe file type to be ready for validation and docking simulations. Validation steps were performed by removing the cocrystallized ligand and redocking it into the binding sites of the selected enzymes. The obtained data is represented as sup. data. Only docking into human SOD enzyme was performed through SwissDock (http://www.swissdock.ch/) by submitting the PDb file and quercetin molecule into the server website.Quercetin3D structure was retrieved from PubChem (https://pubchem.ncbi.nlm.nih.gov/), protonated and subjected to energy minimization by Moe then saved as mdb file to be ready for docking simulations.

#### 2.1.2. Molecular Docking Simulations

**Figure 1:**
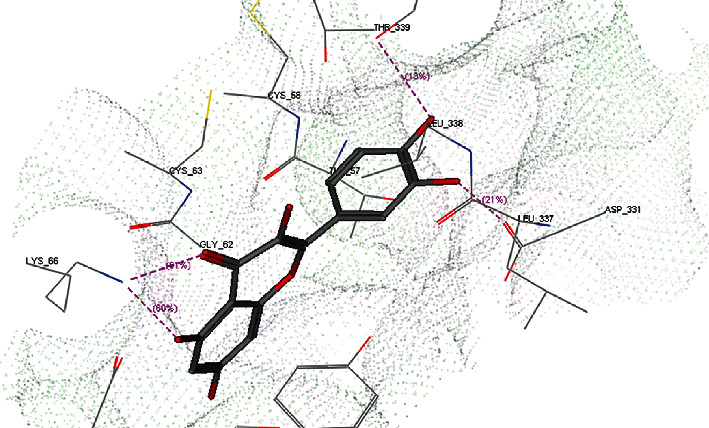
3D binding mode of quercetin with GR (PDb ID: 1BWC).

**Figure 2:**
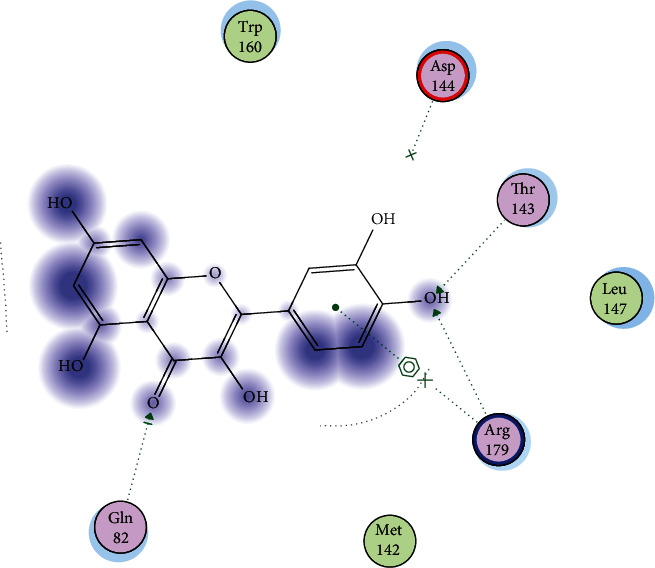
2D interactions of quercetin with GPx enzyme (PDb ID: 2F8A).

**Figure 3:**
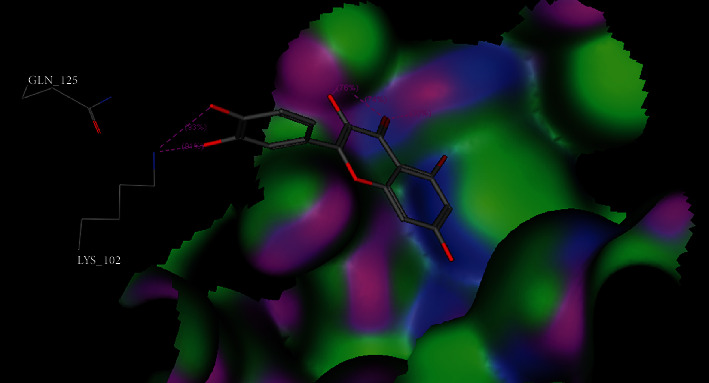
3D binding mode of quercetin inside GST enzyme (PDb ID : 10GS).

**Figure 4:**
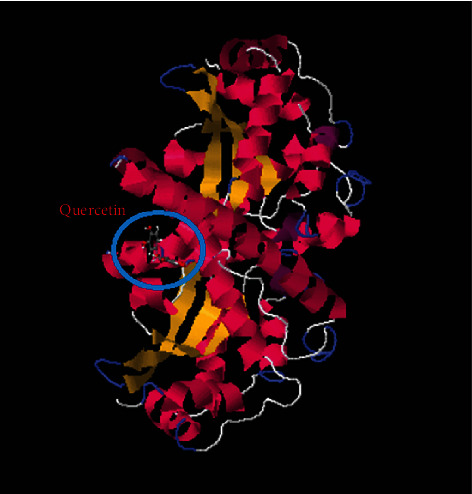
Quercetin docked into human SOD (PDb ID : 1AP5).

**Figure 5:**
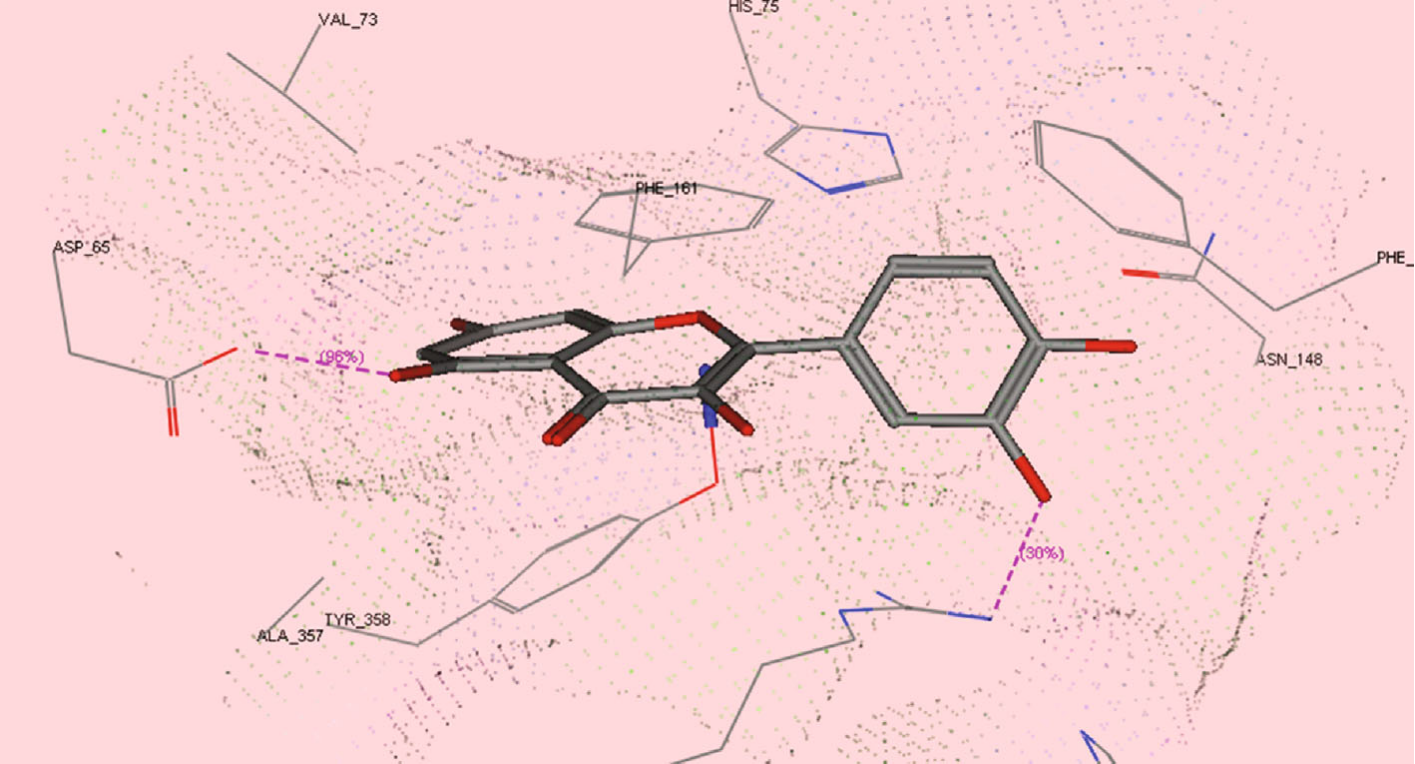
3D binding mode of quercetin inside CAT enzyme (PDb ID : 1DGB).

To detect the binding affinity, mode of binding and possible interactions between our target molecule (quercetin as a ligand) and oxidative stress enzymes (GR, GST, GPx, SOD, and CAT) molecular docking was simulated and the obtained results are represented in Table 1 and Figures 1–5.

### 2.2. Experimental Animal Model

All experiments on animals in this research work were approved from research ethics committee Ain-Shams University REC-ASU (ENREC-ASU. 2020-2). 45 male rats (*Rattusnorvegicus*) with body weight 170-200 g were obtained and divided into three groups, with 15 rats in each group. All groups were allowed two weeks to be acclimated to the new conditions. The three groups are comprised of a control group and a model group (rats raised in a hypoxic chamber where the atmospheric pressure was reduced to obtain an altitude of 5000 m). Rats were allowed to stand for 4 h. (5 days per week) for 4 weeks [27], and the third group that received quercetin (20 mg/kg) of the body weight (through I.P. route) for one month after induction of hypoxia [28]. The brain was removed and placed on an ice-cold glass plate, washed, and dried; samples prepared and collected. Samples used for biochemical analysis was prepared through homogenization then centrifuged at 3000 RPM for 20 min. Samples used for examining histopathological changes of the brain was kept first in 10% formalin and then subjected to fixation and tissue processing.

### 2.3. Biomarker Assessment

Rats from each group were sacrificed, and their brains were removed to be subjected to homogenization in 10 mM Tris–HCl, pH 7.4, with 10 microliter (ml) protease inhibitor cocktail and then centrifuged. The supernatant was used to detect GR, GST, GPx, SOD, CAT, and malondialdehyde (MDA) levels.

#### 2.3.1. Glutathione (GSH) Assessment

GSH was examined as a nonenzymatic antioxidant. PMS (postmitochondrial supernatant) was precipitated with sulphosalicylic acid (4.0%) at a ratio of 1 : 1. The samples were kept at 4°C for 1 h and then subjected to centrifugation at 1200 g for 15 min at 4°C. The assay mixture contained 0.4 ml of supernatant, 2.2 ml of 0.1 M sodium phosphate buffer (pH 7.4), and 0.4 ml DTNB (5,5-dithio-bis-(2-nitrobenzoic acid) (1.0 mM), giving a total volume of 3 ml. The optical density of the reaction product was detected immediately at 412 nm, and the results are expressed as micromol GSH per mg protein [29].

#### 2.3.2. Glutathione Reductase (GR) Assessment

The reaction mixture consisted of phosphate buffer (0.1 M, pH 7.6), NADPH (0.1 mM), EDTA (0.5 mM), oxidized glutathione (1 mM), and 0.05 ml of PMS in a total volume of 1 ml. The enzyme activity was quantified at room temperature by measuring the disappearance of NADPH at 340 nm and calculated as nmol NADPH oxidized/min/mg protein using a molar extinction coefficient of 6.22 × 10^3^ M^−1^ cm^−1^ [30].

#### 2.3.3. Glutathione Peroxidase (GPx) Assessment

Enzyme activity was measured in a mixture consisting of phosphate buffer (0.05 M, pH 7.4), EDTA(1 mM), sodium azide (1 mM), glutathione reductase (1 EU/ml), glutathione (1 mM), NADPH (0.2 mM), hydrogen peroxide (0.25 mM), and 0.1 ml PMS in a final volume of 1 ml. The disappearance of NADPH at 340 nm was recorded at room temperature. The enzyme activity was calculated as nmol NADPH oxidized/min/mg protein using a molar extinction coefficient of 6.22 × 10^3^ M^−1^ cm^−1^ [31].

#### 2.3.4. Glutathione S-Transferase (GST) Assessment

To examine GST activity, 1.45 ml phosphate buffer (0.1 M, pH 6.5) was added to 0.15 ml CDNB (chloro-2,4-dinitrobenzene) (1 mM), 0.2 ml GSH (1 mM), and 0.2 ml PMS of the brain in a cuvette, and the optical density at 340 nm was measured. The enzyme activity was calculated as nmol CDNB conjugate formed/min/mg protein using a molar extinction coefficient of 9.6 × 10^3^ M^−1^ cm^−1^ [32].

#### 2.3.5. Superoxide Dismutase (SOD) Assessment

SOD level was determined by adding 25 ml of supernatant obtained from the centrifuged brain homogenate to 1.2 ml of 0.052 M sodium pyrophosphate buffer (pH 8.3), 0.1 ml of 186 mM phenazonium methiosulphate, 0.3 ml of 300 mM nitroblue tetrazolium, and 0.2 ml of 780 mM NADH. Reaction mixture was incubated for 90 s at 30°C, and the reaction was stopped through the addition of 0.1 ml of glacial acetic acid. The changes in absorbance of the reaction mixture were measured at 560 nm by using spectrophotometer and expressed as U/mg protein. SOD is an enzyme that catalyzes the dismutation of superoxide into oxygen and hydrogen peroxide [33].

#### 2.3.6. Catalase (CAT) Assessment

CAT activity was measured by adding 0.1 ml of supernatant obtained from the homogenized brain tissue to a cuvette containing 1.9 ml of 50 mM phosphate buffer. To this mixture,1.0 ml of freshly prepared 30 mM H_2_O_2_ was added and changes in absorbance for 3 min at 240 nm at an interval of 30 s were measured [34].

#### 2.3.7. Malondialdehyde (MDA) Assessment

MDA was measured by adding 0.2 ml of brain homogenate that was treated with 0.2 ml of sodium dodecyl sulphate (8.1%), 20% of 1.5 ml of acetic acid (pH 3.5), and 1.5 ml of thiobarbituric acid (0.8%). The mixture was made up to 5 ml using distilled water and then heated at 95°C in an oil bath for 60 min. The mixture was cooled and 5 ml of n-butanol and pyridine mixture (15 : 1 *v*/*v*) was added. The mixture was shaken vigorously. After centrifugation at 4000 RPM for 10 min, the organic layer was obtained, and the absorbance at 532 nm was measured [35].

### 2.4. Histopathological Examination

The second half of the brain was transferred to 10% formalin and underwent fixation and tissue processing. The formalin-preserved rats' brain tissue specimens were processed using an automated tissue processor. The process consisted of an initial two steps that are fixation and dehydration. Fixation comprised tissue immersion in 10% buffered formalin for 48 h, followed by removal of the fixative then immersion in distilled water for 30 min. Dehydration was then carried out by immersing the tissues through a graded series of alcohol (70%, 90%, and 100%). The tissue was initially exposed to 70% alcohol for 120 minutes, followed by 90% alcohol for 90 minutes and then two cycles of absolute alcohol, one hour for each one. Dehydration was followed by clearing the samples using several changes of xylene. Tissue was immersed for an hour in a mixture of 50% alcohol and 50% xylene, followed by immersion in pure xylene for 1.5 h. These tissues were then impregnated with molten paraffin wax, embedded, and blocked. Paraffin sections (4–5 *μ*m) were stained with hematoxylin and eosin. The stained sections were examined for normal histomorphological structures, in addition to signs of circulatory disturbances, inflammation, degeneration, apoptosis, necrosis, and any other pathological changes in the examined tissues [36].

### 2.5. Statistical Analysis

The obtained results are expressed as mean ± standard deviation. Biochemical and behavioral parameters were compared using a one-way analysis of variance. *P* < 0.005 was considered statistically significant. Statistical analysis was performed using SPSS (version 22).

## 3. Results

Cerebral damage is a complicated health problem that if not treated may leave a permanent effect on the other body organs. High altitudes represent a common major cause for cerebral damage and overexpression of oxidative stress enzymes are usually noticed. In this research work, we have decided to use CADD (computer-aided drug design) to explore the ability of quercetin to target the oxidative stress enzymes, the results revealed promiscuity of quercetin to bind to these enzymes. The next step was to explore this effect in animals to see how much quercetin is able to affect oxidative stress enzyme levels; additionally, histopathological examinations are also considered in this research work.

### 3.1. Molecular Docking Studies

For GR, quercetin showed better affinity to GR enzyme than the cocrystallized ligand flavin adenine dinucleotide (FAD); their docking score energy is -19.75 Kcal/mol and -8.93 Kcal/mol, respectively (Table 1). Moreover, quercetin showed amino acid interactions with Ser30, Asp331, and Lys66 through hydrogen bonding with 3 quercetin hydroxyl groups and another fourth hydrogen bond with Lys66 through the oxygen atom of the quercetin carbonyl group (Figure 1 and sup. Data(available here)).

For GPX, after performing validation step and redocking of the cocrystallized ligand into human glutathione peroxidase enzyme (PDb ID: 2F8A), the obtained data revealed that the affinity of quercetin to GPx is better than the crystallized ligand as the score energy was -14.66 and -6.72 Kcal/mol, respectively, as shown in Table 1. Also, quercetin showed good interactions with GPx amino acids as it has showed ability to form three hydrogen bonding with Thr143, Arg179, and Gln82. Additionally, quercetin also has showed ability to form arene-cation interaction through the phenyl moiety with Arg179 (Figure 2 and Sup. Data).

For GST, docking results of quercetin GST binding site (PDb ID : 10GS) showed that the affinity of quercetin to bind into human GST is closely near to that of L-gamma-glutamyl-S-benzyl-N-[(S)-carboxy(phenyl)methyl]-L-cysteinamide (cocrystallized ligand) as presented in Table 1. The binding score energy for quercetin and the crystallized ligand is -12.94 and -14.23 Kcal/mol, respectively. Moreover, quercetin showed ability to form five hydrogen bonding interactions through its hydroxyl groups and oxygen atom of the carbonyl group with GST amino acids LysB102 (2 hydrogen bonds), LysA44 (2 hydrogen bonds), and TrpA38 (1 hydrogen bond) as shown in Figure 3 and sup. Data.

For SOD, SwissDock server (http://www.swissdock.ch/docking) was used to perform docking of quercetin into human SOD binding site; both quercetin and SOD pdb file (1AP5) were uploaded into SwissDock server as pdb and mol2 files, respectively. The obtained results revealed that quercetin can bind into human SOD enzyme (PDb ID : 1AP5) with fullfitness value equal to -2431.18 Kcal/mol and estimated Δ*G* value equal to -7.76 (Sup. Data).  Figure 4 is showing the docked quercetin molecule with SOD enzyme as obtained from SwissDock server (Why did this value not show in Table 1).

For CAT, quercetin showed ability to form 2 hydrogen bonds between the hydroxyl groups and ArgB354 and AspD65 amino acids. In addition to two arene-arene interactions between the phenyl moieties of quercetin and TyrB358, PheB153 amino acids (Figure 5) were also observed. Also, as for affinity of quercetin towards human CAT enzyme (PDB ID : 1DGB) was found to be substantially good when compared with protoporphyrin IX which is the cocrystallized ligand, the docking score shown in Table 1 is -16.60 and -23.83 Kcal/mol, respectively.

### 3.2. Biomarker Assessment

GSH, GR, GPX, GST, SOD, and CAT levels in the model group were significantly lower than those in the control group. However, the levels of GSH, GR, GPX, GST, SOD, and CAT in the quercetin-treated group were significantly higher (*P* < 0.05). The MDA level was lower in the model group and in quercetin-treated group, however, it showed lower level in the model group (*P* < 0.05).

### 3.3. Histopathological Findings

**Figure 6:**
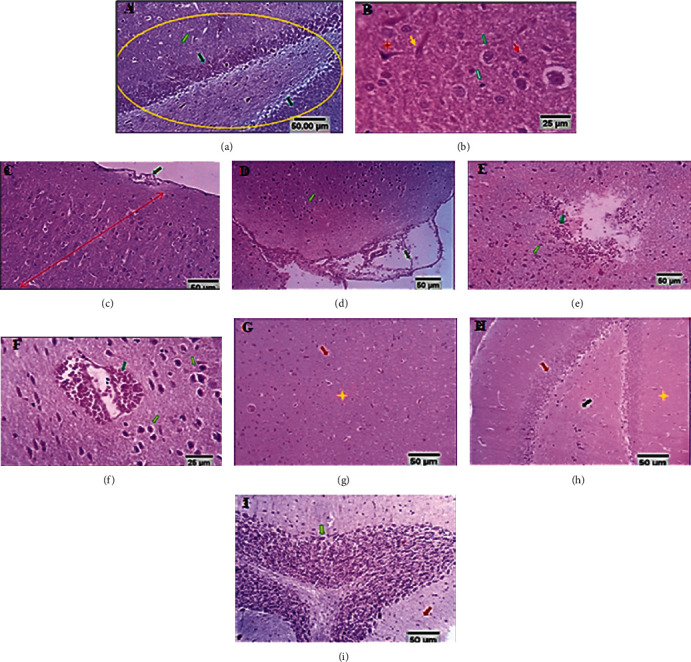
(a–c) Brain of control rats (scale bars, 50 *μ*m, 25 *μ*m). (d–f) Brain tissue of model group rats (Scale bars 50 *μ*m, 25 *μ*m). (g–i) Brain tissue of the quercetin-treated group (scale bars, 50 *μ*m, 25 *μ*m).

Figures 6(a)–6(c) represent the brain of control rats with normal hippocampal histology (yellow circle) comprising pyramidal astrocytes and nerve fibers (yellow and black arrows). Cerebral cortical tissue containing a normal arrangement of the six layers (red double arrow) comprised of the large neurons (green arrow), pyramidal fusi form neurons (yellow arrow), oligodendroglia (red arrow), and microglia (gray arrow). Cerebellar tissue showing the normal molecular (yellow star) and granular layers (brown star). Figures 6(d)–(f)) exhibit brain tissue of model rats with characteristic vascular changes represented by meningocerebral vascular dilatation, congestion, and multifocal hemorrhages associated with widespread neuronal degeneration; it is also showing focal interstitial hemorrhage and widespread neuronal degeneration. Neuronal degeneration is characterized by the disruption of the normal arrangement of cell layers, cells are bigger in size with perineural edema and large vascular spaces around them and the myelin sheath around degenerated oligodendroglial cells of white matter appeared vacuolated (partial demyelination). Figures 6(g)–(i)) show the brain tissue of the quercetin-treated group with well-known normal structures of the cerebral cortex, hippocampus. Mild focal cerebellar neuronal degeneration and white matter nerve fibers demyelination.

**Figure 7:**
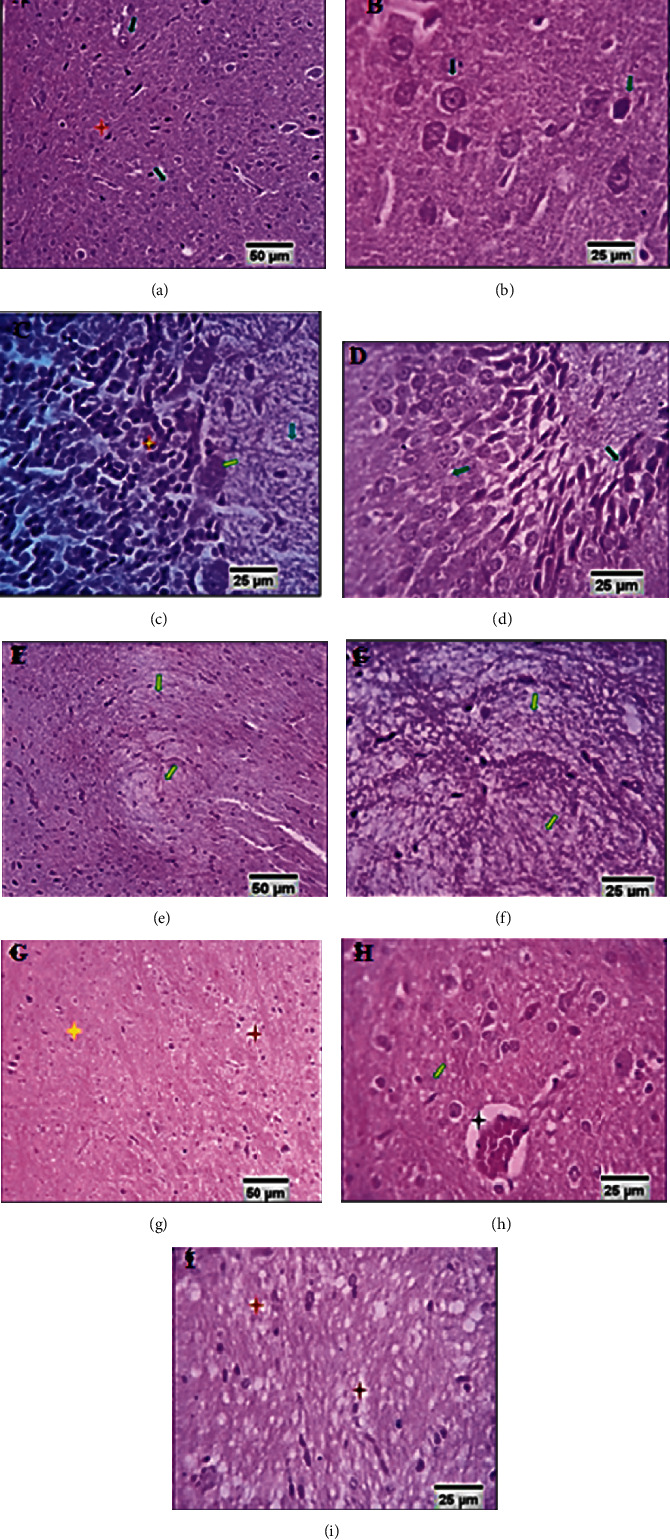
(a–c) Brain tissue of control rats (scale bars, 50 *μ*m, 25 *μ*m). (d–f) Brain tissue of model rats (scale bars, 50 *μ*m, 25 *μ*m). (g–i) Brain tissue of the quercetin-treated group (scale bars, 50 *μ*m, 25 *μ*m).

Figures 7(a)–(c) is exhibiting the brain tissue of control rats, including cerebral and cerebellar structures comprising normal large neurons (black arrows), fusiform neurons (green arrow), and molecular nerve fibers (blue arrow). Figures 7(d)–7(e) is showing the brain tissue of the model rats showing hippocampal reactive astrocytosis (1, blackarrow), focal cerebral astrocytic-large neuronal proliferation (reverse-replicative reaction; red stars), and neurotoxic axonal degeneration and demyelination (gray arrows and yellow stars). Also, the brain tissue of model rats' cerebella shows focal cell degeneration, necrosis, and complete losses, alongside disruption of the granular cell layer. Figures 7(g)–7(i) represent the brain tissue of the quercetin-treated group, and the six layers from superficial to deep were found to be clear; common cells inside these layers were neurons, especially pyramidal, granule cells and neuroglial cells. The pink-stained background is showing that the neuropil was a mat of neuronal and glial cell. Only few cases were found to show mild focal cerebellar neuronal degeneration and white matter nerve fiber demyelination.

## 4. Discussion

Many people worldwide move from low-altitude locations to higher-altitude locations when traveling or hiking and sometimes fall sick. Therefore, the given study deals with the argumentation of these kinds of sickness using a natural food additive compound, quercetin. However many precautions are taken to prevent and treat high-altitude sickness and reduce the side effects of treatment; the incidence of high-altitude sickness has been increased [37]. High-altitude sickness may be annoying, causing dissatisfaction, lack of appetite, feeling faint, and difficulty of breathing. The main goals for the treatment of high-altitude sickness are to increase oxygen supply or inhibit factors that are responsible for cytokine release and inflammation. However, synthetic chemical compounds cannot fully protect against high-altitude sickness, and there are many side effects of these chemical drugs [38]. Therefore, we should extensively examine natural extracts that can be used instead of chemical drugs to prevent high-altitude sickness. Quercetin is a major constituent of natural supplements commonly used for memory improvement; it can also be taken to reduce high-altitude sickness [39]. The seeds of quercetin are also used to protect against hypoxia resulting from decreased oxygen supply to the brain [40]. Quercetin, is a ubiquitous plant-derived bioflavonoid possesses several beneficial

pharmacological effects, e.g., cardioprotective, antiallergic, and antiproliferative.

In the present study, the use of quercetin as a treatment of high-altitude sickness was examined, and it was found to cause changes in the hypoxiain-treated group of rats. The obtained data illustrated that quercetin has a good effect on antioxidant variables that are related to reduced brain edema. The current protections against brain damage depend on the ability of chemical drugs or natural products to affect free radical scavengers. As it is known that the inhibition of oxidative stress, inflammation, and apoptosis provides neuroprotective effects against cerebral injury, quercetin has both antioxidant and anti-inflammatory effects, against oxidative and inflammatory diseases, Also, evidence indicates that the scavenging of free radicals and inhibition of free radical generation are improved, which is proving quercetin's antioxidant effects [41]. The obtained results in our research work revealed the hopeful effect of quercetin to bind to oxidative stress enzymes, and molecular docking studies showed a negative docking score energies comparable with that of the cocrystallized ligands. Additionally, binding to amino acids through quercetin functional groups was also observed as shown in Figures 1–5 and sup. Data files. The next step in our work was to explore the real activity of quercetin against oxidative stress enzymes expression levels. Biomarker assay results demonstrated a decrease in GSH levels (resulting from increasing utilization of toxic radicals) in the model group [42]. As shown in Table 2, lower levels of SOD were observed in the brains of ischemic rats, demonstrating the role of the superoxide radical in cytotoxicity [43]. The obtained results also showed the ability of quercetin to inhibit SOD activity in rats kept in high-altitude-like conditions. Furthermore, GPx can scavenge free radicals that can cause cell damage [44]. Many studies have confirmed the role of GPx, SOD, and CAT in utilizing the free radicals resulting from H_2_O_2_ [45]. Contrastingly, the level of CAT was lower in the brains of hypoxic rats. Furthermore, in agreement with previous studies [46], the present study found that hypoxia is related to oxidative stress, as observed by increased brain MDA levels. Increased levels of MDA that represent an indication of brain damage induced by toxic lipid peroxidation, and the decreased MDA levels in the quercetin group were an indicator for the protective role of quercetin against the toxicity in the brains of rats. Quercetin supplementation in the treated group decreased the MDA levels in the brain tissue [47, 48]. The results demonstrated the positive effects of quercetin against brain damage. The protective effects of quercetin have also been observed in cultured neurons that provided neuroprotection when given early in the course of the disease. This neuroprotective role was reported to be a result of the intervention of iron induced-oxidative stress-dependent apoptotic pathways [12]. Furthermore, the obtained results illustrate that quercetin has the ability to enhance levels of GR, GPx, and GST to inhibit ROS activity (Table 2). The obtained results showed that MDA was reduced by quercetin and showed also an increase in SOD levels. The other antioxidant enzymes were found to be around normal levels upon treatment with quercetin. This indicates that quercetin is a good antioxidant agent and its activity is due to its ability to scavenge free radicals, reduce lipid peroxidation and superoxide radical formation, and can also increases glutathione and glutathione peroxidase levels [49, 50]. Therefore consistent with these results, in the present study, quercetin reversed the hypoxia-induced increase in MDA and decrease in GPx, GSH, and SOD.

The histopathological examination revealed that rats exposed to a high-altitude, low-pressure, and low oxygen environment had neuronal degeneration, characterized by disruption of the normal arrangement of cell layers, cells became larger in size with large vascular spaces around them, and a myelin sheath around degenerated oligodendroglial cells, with the white matter appearing vacuolated (partial demyelination) (Figure 6). Pathognomonic hippocampal reactive astrocytosis, focal cerebral astrocytic-large neuronal proliferation (reverse-replicative reaction), and neurotoxic axonal degeneration and demyelization were also recorded (Figure 7). Focal Purkinje cell degeneration, necrosis, and complete loss, in addition to disruption of the granular cell layer, were seen in the cerebellum (Figure 7). Microscopic examination of the quercetin group sections found that the cerebral cortex and medulla of different areas, including the hippocampus, cerebellar medulla, molecular granular, and Purkinje cell layers, demonstrated the well-known normal structures.

## 5. Conclusion

Brain damage can occur in high-altitude locations with low pressure and low oxygen, resulting in cerebral edema. Antioxidant assessment demonstrated significant changes in all antioxidant variables in model animals. However, after treatment with quercetin, GSH, GR, GPX, GST, SOD, and CAT levels were found higher, while MDA levels were found to be low. Furthermore, the protective effects were observed in a histopathological study that confirmed the role of quercetin in the treatment of brain damage and its ability to scavenge free radicals resulting from hypoxia. Additionally, molecular docking studies also proved the potential effect of quercetin to target the antioxidative stress enzymes. Finally, from molecular docking studies, biomarkers level detection and in vivo studies we can say that it is recommended for people living in high altitudes to take quercetin rich diets to reduce symptoms that may appear from hypoxia and oxidative stress enzyme elevation.

## Figures and Tables

**Table 1:** Docking score energies for quercetin and the cocrystallized ligands inside antioxidative stress enzymes.

	GR	GPX	GST	CAT	SOD
Quercetin	-19.75 Kcal/mol	-14.66 Kcal/mol	-12.94 Kcal/mol	-16.60 Kcal/mol	-2431.18 Kcal/mol
Cocrystallized ligand	-8.93 Kcal/mol	-6.72 Kcal/mol	-14.23 Kcal/mol	-23.83 Kcal/mol	-7.76 Kcal/mol

**Table 2:** Biomarker analysis of brain tissues.

	GSH (U/mg)	GR (U/mg)	GPX (U/mg)	GST (U/mg)	SOD (U/mg)	CAT (U/mg)	MDA (U/mg)
Control group	9.8 ± 0.27	3.05 ± 0.14	1.54 ± 0.08	16.75 ± 0.94	963.24 ± 3.87	5.31 ± 0.17	21.6 ± 1.14
Model group	5.31 ± 0.17	0.78 ± 0.57	0.23 ± 0.003	2.49 ± 0.13	238.18 ± 2.71	1.27 ± 0.06	31.14 ± 1.36
Quercetin group	8.5 ± 0.23^∗^	2.71 ± 0.13^∗^	1.46 ± 0.07^∗^	15.87 ± 0.87^∗^	879.52 ± 3.12^∗^	4.15 ± 0.15∗	22.37 ± 1.05^∗^

^∗^ *P* < 0.05 significant difference from the model group (*n* = 15/group).

## Data Availability

All supporting data are included in the supplementary data files.

## Supplementary Materials

Supplementary Materials Supporting data files: alongside with this manuscript we have incorporated the other supporting figures for validation steps, 2D and 3D interactions with different enzymes are also incorporated. They are provided in five separate files each file for a specific type of our selected molecular targets.
