# Supplementary material for: Biological Effect of Quercetin in Repairing Brain Damage and Cerebral Changes in Rats: Molecular Docking and In Vivo Studies
Source: Biomed Res Int. 2022 Apr 26;2022:8962149. doi: 10.1155/2022/8962149 (PMC9071882; doi:10.1155/2022/8962149)
Supplement: Supplementary Materials — Supporting data files: alongside with this manuscript we have incorporated the other supporting figures for validation steps, 2D and 3D interactions with different enzymes are also incorporated. They are provided in five separate files each file for a specific type of our selected molecular targets. [file 8962149.f1.zip › 2D and 3D figures for docking with GST.pdf]

2D and 3D figures for docking with GST

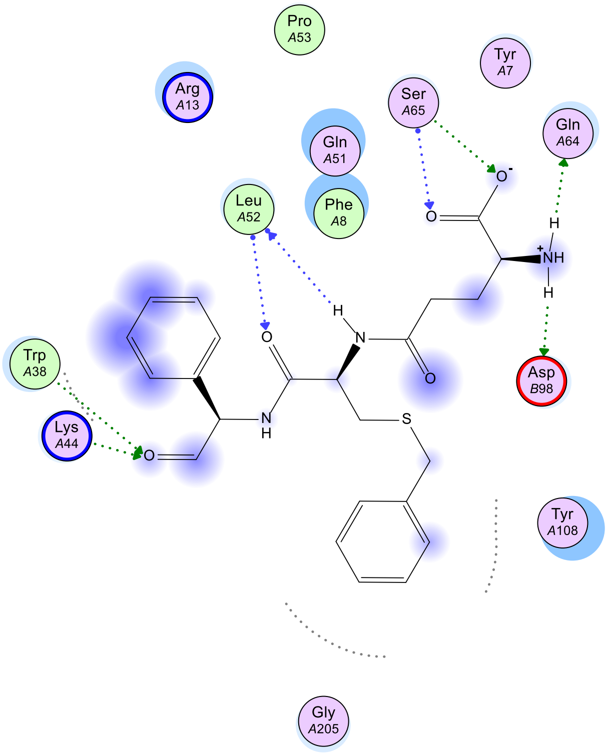

Co-crystallized ligand (**L-gamma-glutamyl-S-benzyl-N-[(S)-carboxy(phenyl)methyl]-L-cysteinamide**) inside 10GS, S = -14,23 Kcal/mol

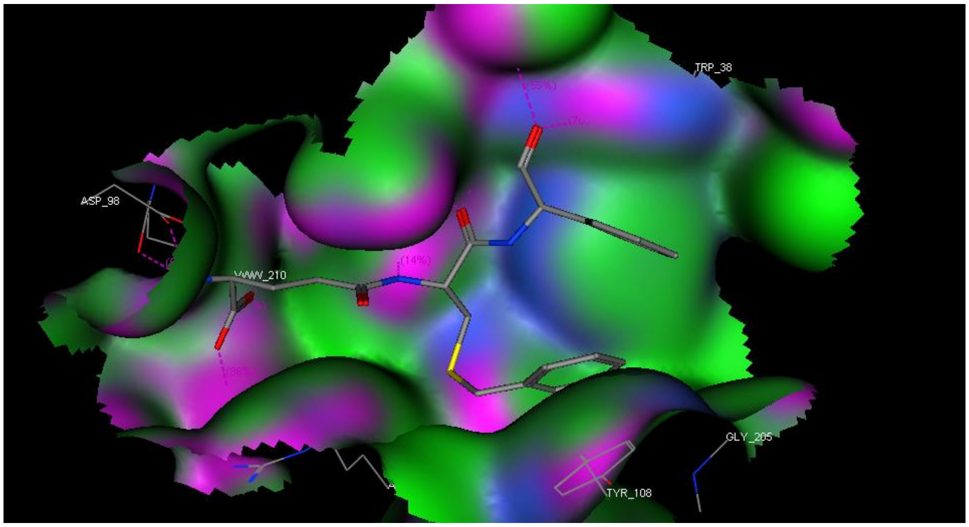

Co-crystallized ligand (**L-gamma-glutamyl-S-benzyl-N-[(S)-carboxy(phenyl)methyl]-L-cysteinamide**) inside 10GS, S = -14,23 Kcal/mol

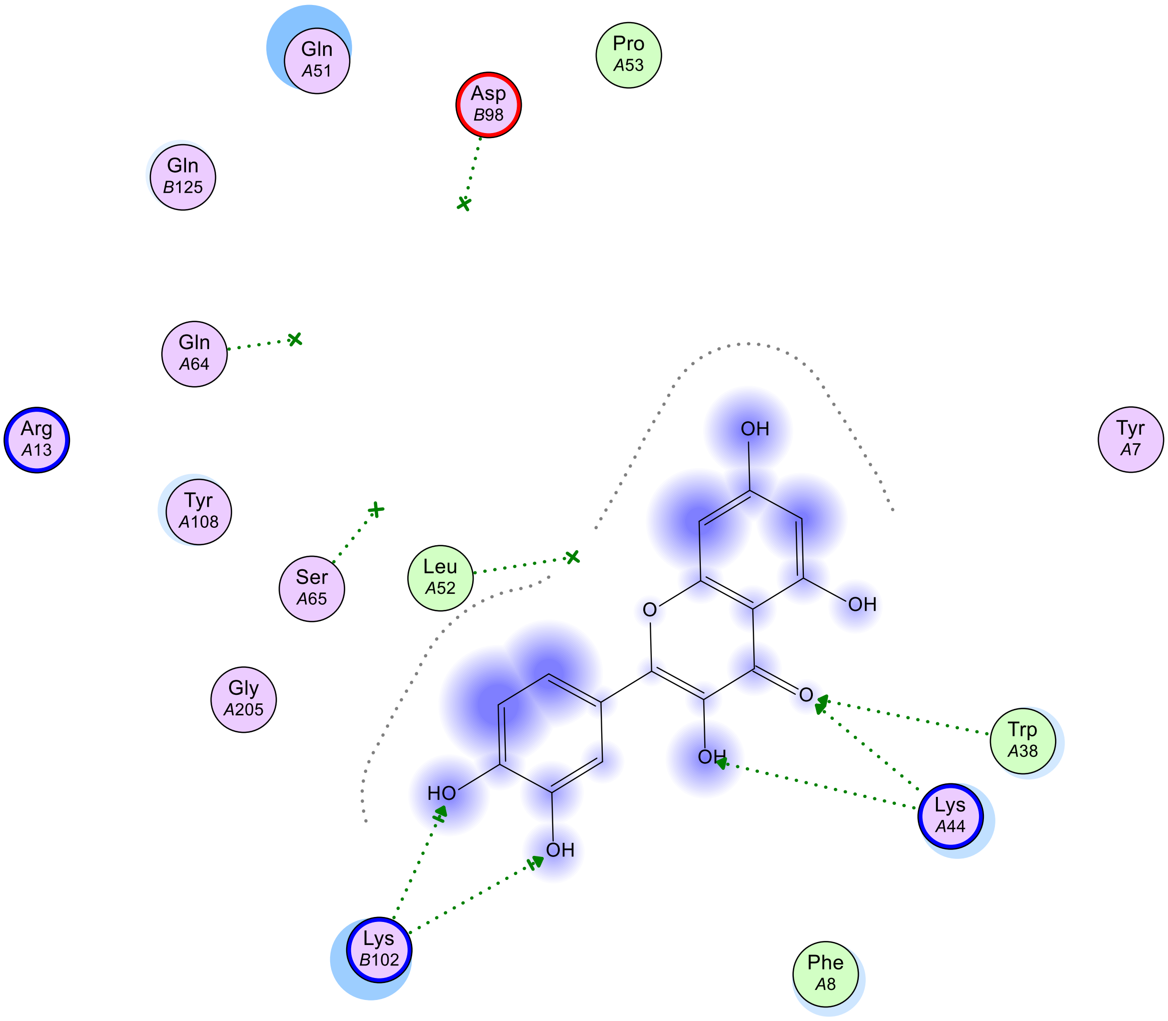
Gln
A51
Pro
A53
Asp
B98
Gln
B125
Gln
A64
Arg
A13
Tyr
A7
Tyr
A108
Ser
A65
Leu
A52
OH
OH
O
Gly
A205
O
Trp
A38
OH
HO
Lys
A44
OH
Lys
B102
Phe
A8

Quercetin

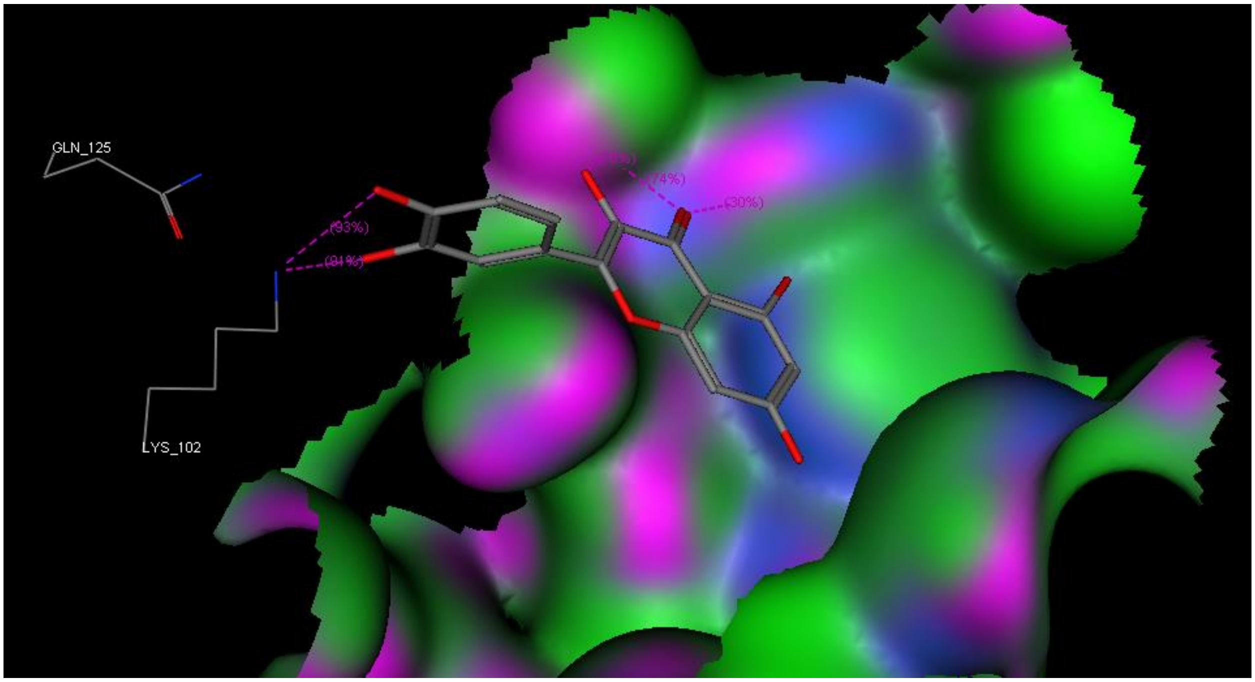
GLN_125
(74%)
(30%)
(93%)
(81%)
LYS_102
