# Supplementary material for: Biological Effect of Quercetin in Repairing Brain Damage and Cerebral Changes in Rats: Molecular Docking and In Vivo Studies
Source: Biomed Res Int. 2022 Apr 26;2022:8962149. doi: 10.1155/2022/8962149 (PMC9071882; doi:10.1155/2022/8962149)
Supplement: Supplementary Materials — Supporting data files: alongside with this manuscript we have incorporated the other supporting figures for validation steps, 2D and 3D interactions with different enzymes are also incorporated. They are provided in five separate files each file for a specific type of our selected molecular targets. [file 8962149.f1.zip › 2D and 3D for docking with GR.pdf]

2D and 3D figures for docking with GR

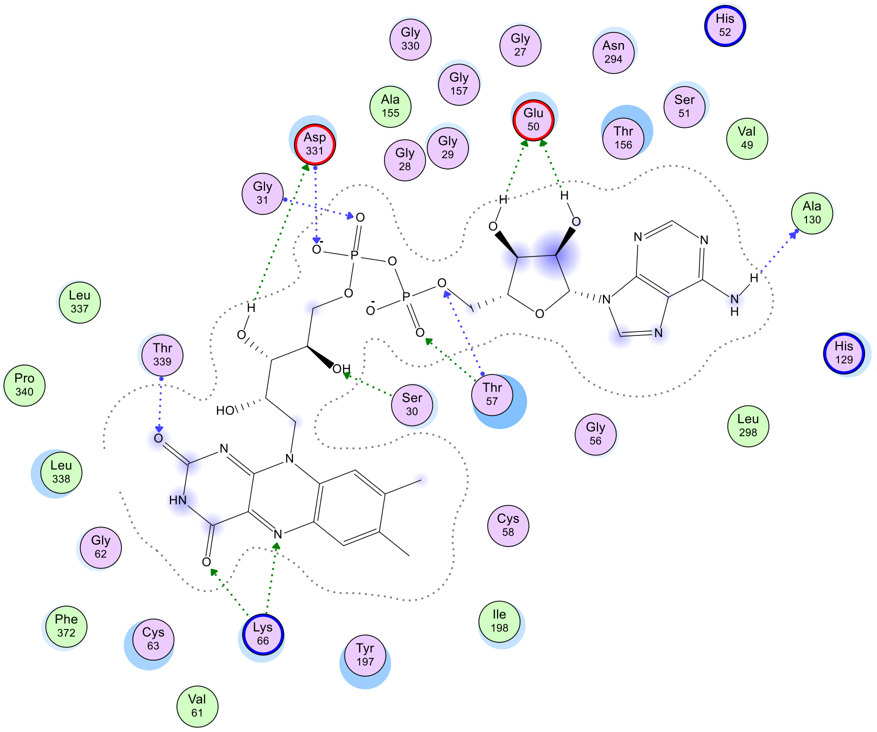

Co-crystallized ligand inside 1BWC , S = - 8.93 Kcal/mol

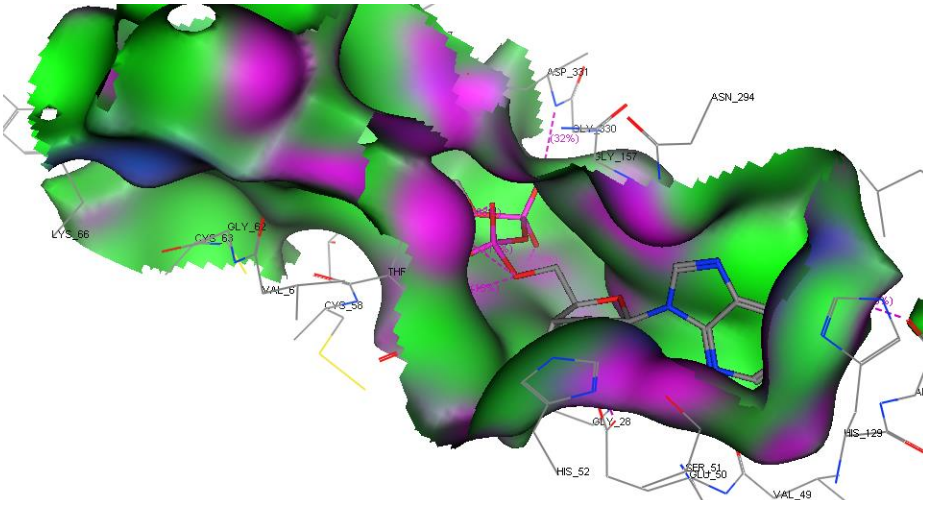

Co-crystallized ligand inside 1BWC

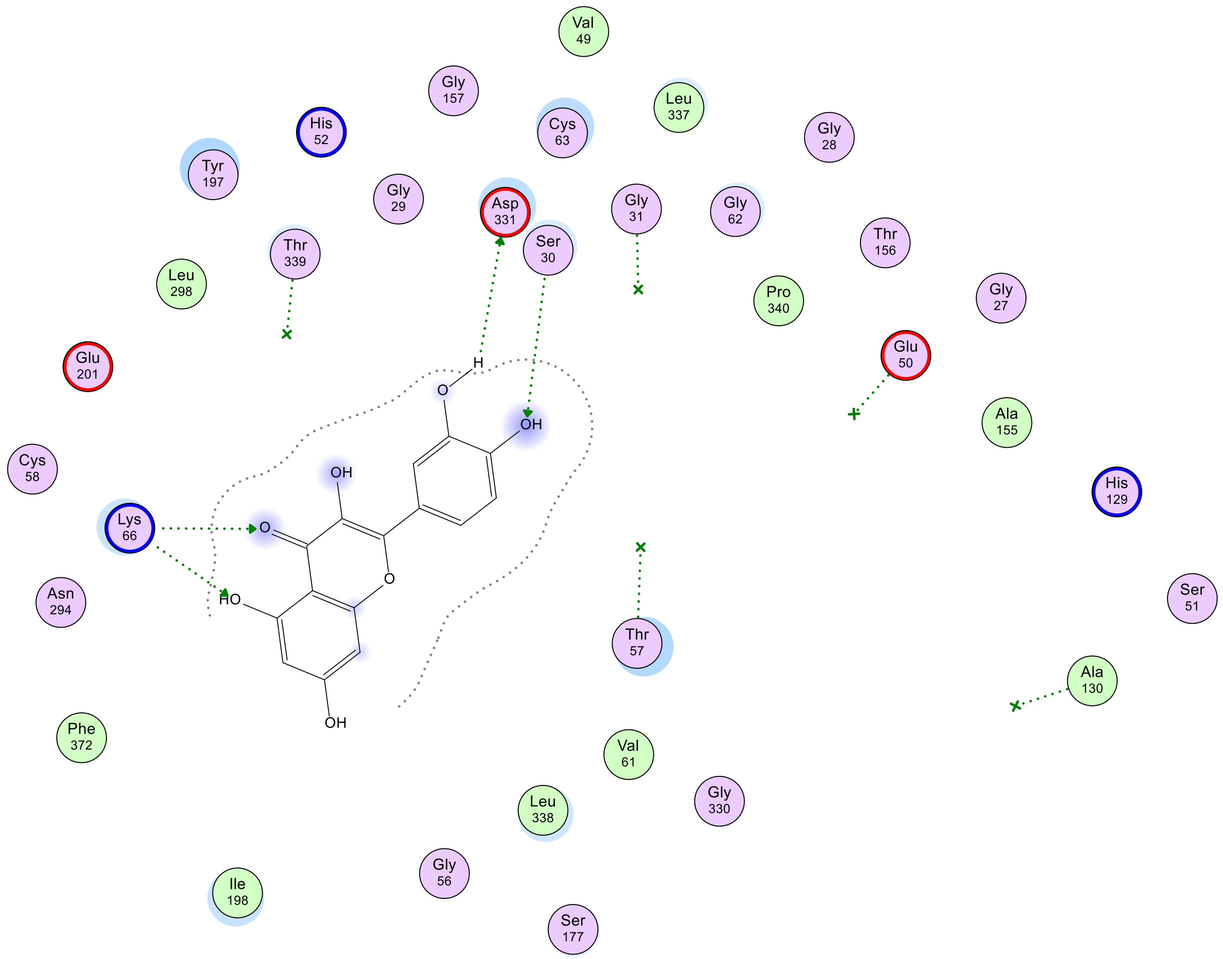

2D interactions of quercetin

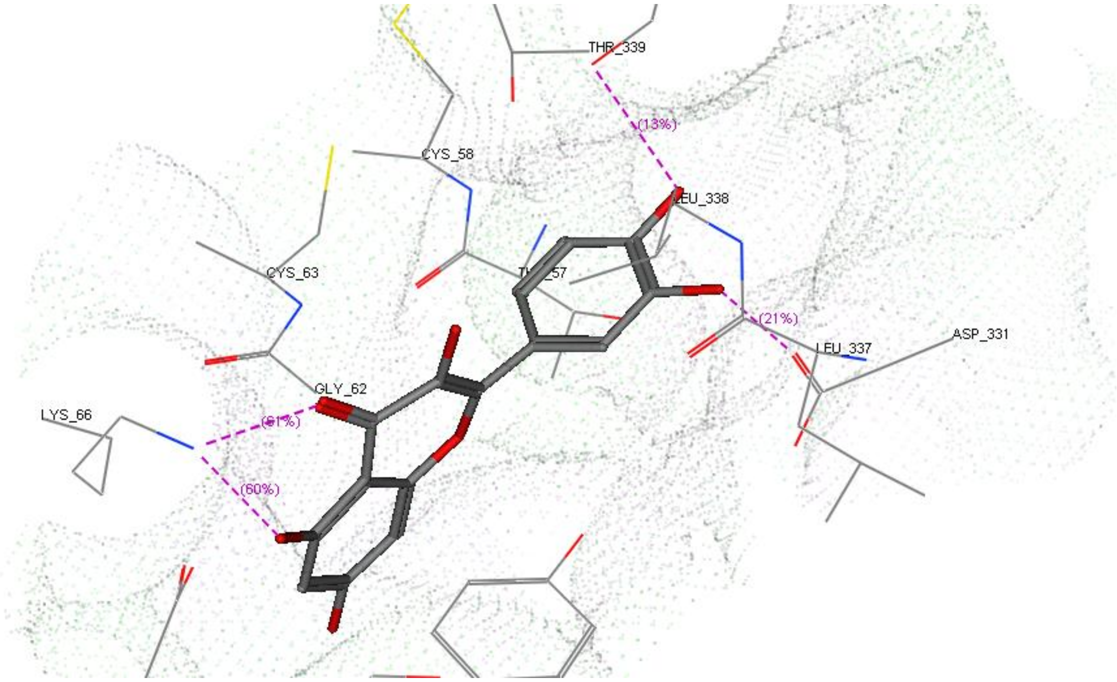

3D Quercetin
