# Supplementary material for: Biological Effect of Quercetin in Repairing Brain Damage and Cerebral Changes in Rats: Molecular Docking and In Vivo Studies
Source: Biomed Res Int. 2022 Apr 26;2022:8962149. doi: 10.1155/2022/8962149 (PMC9071882; doi:10.1155/2022/8962149)
Supplement: Supplementary Materials — Supporting data files: alongside with this manuscript we have incorporated the other supporting figures for validation steps, 2D and 3D interactions with different enzymes are also incorporated. They are provided in five separate files each file for a specific type of our selected molecular targets. [file 8962149.f1.zip › 2D and 3D with CAT.pdf]

2D and 3D figures for Docking with CAT

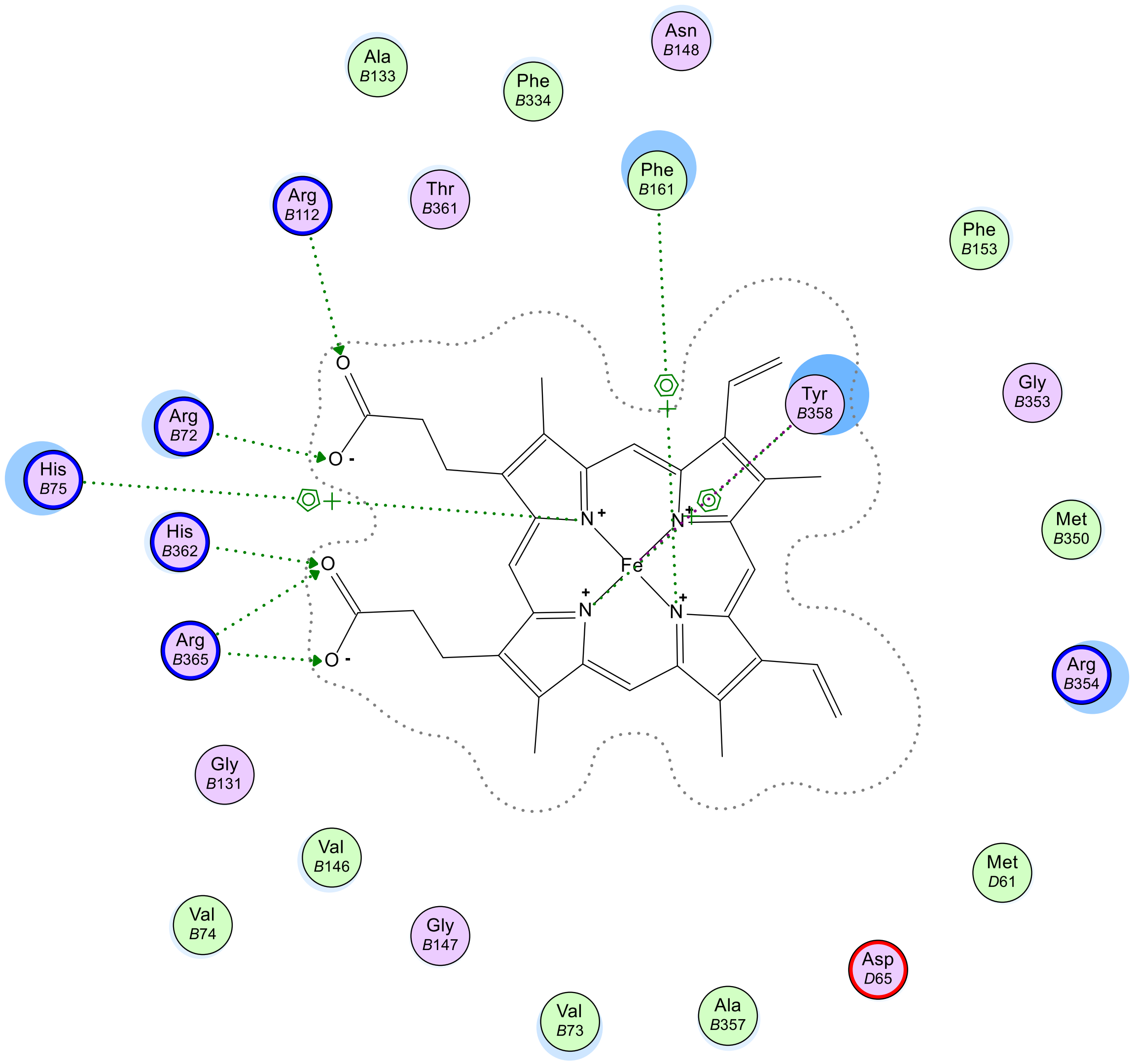

The co-crystallized ligand (**PROTOPORPHYRIN IX CONTAINING FE)**

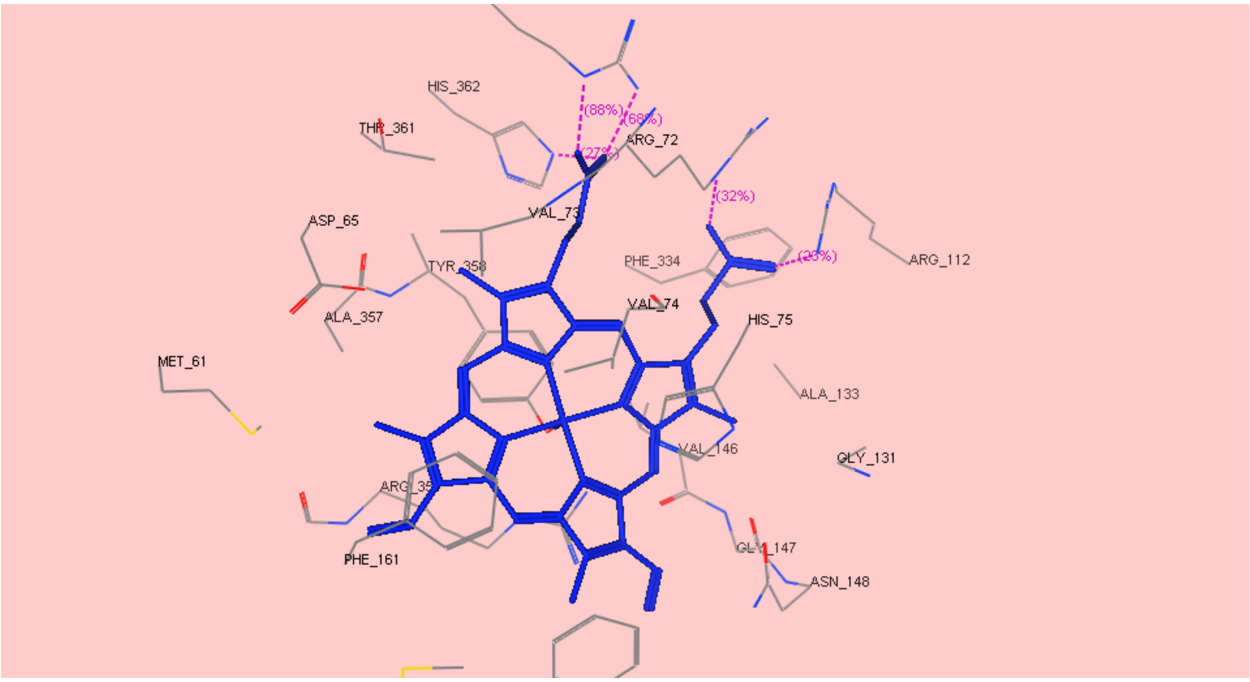

The co-crystallized ligand (**PROTOPORPHYRIN IX CONTAINING FE) (Blue colored)**

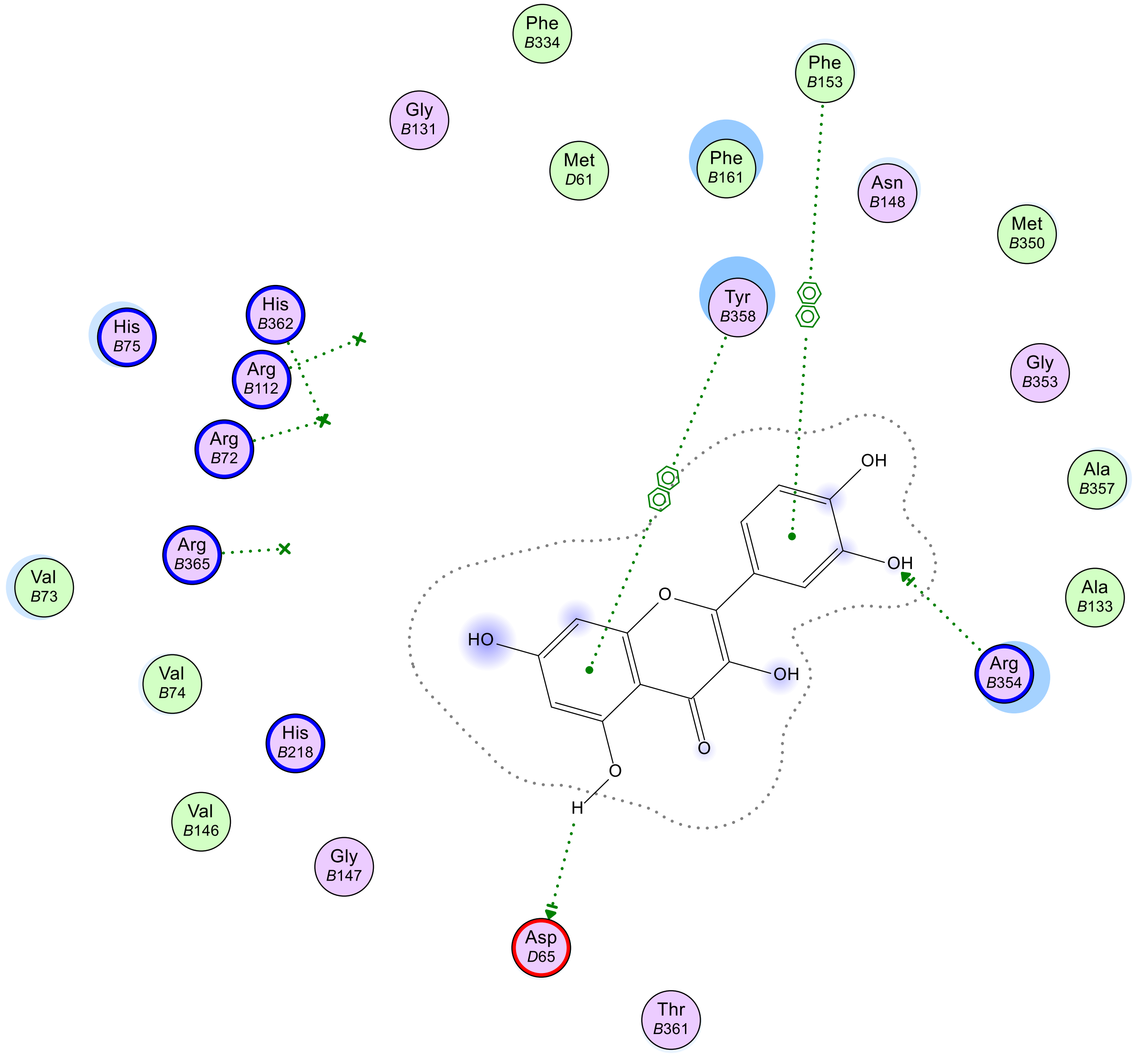

Phe
B334
Phe
B153
Gly
B131
Met
D61
Phe
B161
Asn
B148
Met
B350
His
B75
His
B362
Arg
B112
Arg
B72
Tyr
B358
Gly
B353
Ala
B357
Arg
B365
Val
B73
Ala
B133
Arg
B354
Val
B74
His
B218
Val
B146
Gly
B147
Asp
D65
Thr
B361

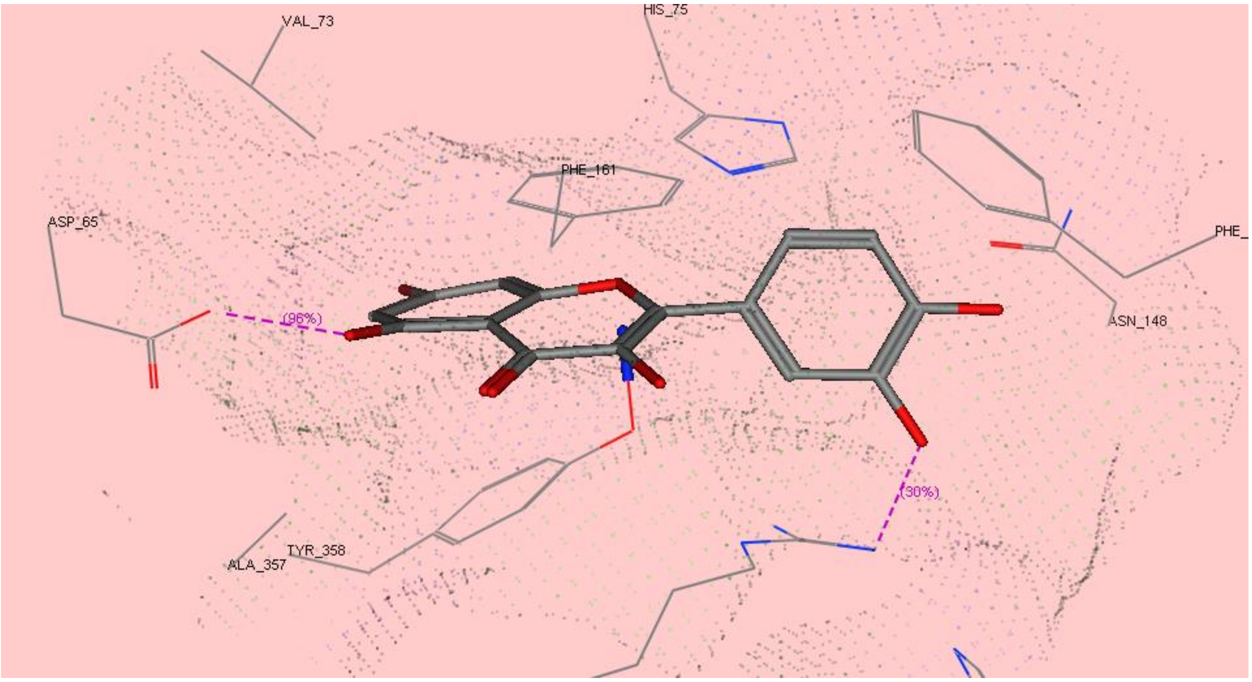
VAL_73
HIS_75
PHE_161
ASP_65
(96%)
ASN_148
(30%)
TYR_358
ALA_357
